# Sacubitril/Valsartan vs. Standard Medical Therapy on Exercise Capacity in HFrEF Patients

**DOI:** 10.3390/life13051174

**Published:** 2023-05-12

**Authors:** Alfonso Campanile, Valeria Visco, Stefania De Carlo, Germano Junior Ferruzzi, Costantino Mancusi, Carmine Izzo, Felice Mongiello, Paola Di Pietro, Nicola Virtuoso, Amelia Ravera, Domenico Bonadies, Carmine Vecchione, Michele Ciccarelli

**Affiliations:** 1 Cardiology Unit, University Hospital “San Giovanni di Dio e Ruggi d’Aragona”, 84131 Salerno, Italy; 2 Department of Medicine, Surgery and Dentistry, University of Salerno, 84081 Baronissi, Italy; 3 Department of Medicine and Surgery, University of Perugia, 06123 Perugia, Italy; 4 Department of Advanced Biomedical Sciences, Federico II University of Naples, 80138 Naples, Italy; 5 Vascular Physiopathology Unit, IRCCS Neuromed, 86077 Pozzilli, Italy

**Keywords:** sacubitril/valsartan, heart failure, HFrEF, CPET, exercise capacity, peak VO_2_

## Abstract

Sacubitril/valsartan (Sac/Val) reduces mortality in patients with heart failure with reduced ejection fraction (HFrEF) compared to enalapril. However, its effects on functional capacity remain uncertain; consequently, we sought to compare Sac/Val vs. standard medical therapy, in terms of effects on prognostically significant CPET parameters, in HFrEF patients during a long follow-up period. We conducted a single-center, observational study in an HF clinic; specifically, we retrospectively identified that 12 patients switched to Sac/Val and 13 patients that managed with standard, optimal medical therapy (control group). At each visit, baseline, and follow-up (median time: 16 months; IQ range: 11.5–22), we collected demographic information, medical history, vital signs, cardiopulmonary exercise testing, standard laboratory data, pharmacological treatment information, and echocardiographic parameters. The study’s primary end-point was the change from baseline in peak VO_2_ (adjusted to body weight). We did not observe significant differences between the two study groups at baseline. Similarly, we did not observe any significant differences during the follow-up in mean values of peak VO_2_ corrected for body weight: Sac/Val baseline: 12.2 ± 4.6 and FU: 12.7 ± 3.3 vs. control group: 13.1 ± 4.2 and 13.0 ± 4.2 mL/kg/min; *p* = 0.49. No significant treatment differences were observed for changes in VE/VCO_2_ slope: Sac/Val baseline: 35.4 ± 7.4 and FU: 37.2 ± 13.1 vs. control group: 34.6 ± 9.1 and 34.0 ± 7.3; *p* = 0.49. In conclusion, after a median follow-up period of 16 months, there was no significant benefit of Sac/Val on peak VO_2_ and other measures of CPET compared with standard optimal therapy in patients with HFrEF.

## 1. Introduction

Sacubitril/Valsartan (Sac/Val) significantly reduced heart failure (HF) hospitalizations and mortality in the PARADIGM-HF trial [1]. It also showed an essential role in improving the quality of life in patients with chronic HF [2,3]. However, its effects on functional capacity remain uncertain, with controversial results emerging from the studies currently available in the literature [4,5,6].

The cardiopulmonary exercise test (CPET) is a valuable tool in HF with reduced ejection fraction (HFrEF). CPET allows the assessment of relevant parameters for functional capacity and prognostic evaluation (e.g., peak VO_2_ and minute ventilation/carbon dioxide production relationship [VE/VCO2 slope]) [7,8,9,10,11,12,13].

The previous studies investigating the Sac/Val effects on cardiopulmonary testing parameters are mainly characterized by a short-term follow-up (3–6 months).

On this clinical background, we evaluated prognostically significant CPET parameters in a population with chronic HFrEF and its effect on prognosis, comparing Sac/Val vs. standard medical therapy in a more extended follow-up period.

## 2. Materials and Methods

### 2.1. Study Design and Participants

We conducted a single-center, observational study in a HF clinic of a tertiary cardiac center in Italy (Salerno). We retrospectively identified 12 patients who switched to Sac/Val, according to the European guidelines [14], and 13 patients managed with standard, optimal medical therapy (control group). With this sample size, assuming a study power of 80%, with a 2-sided type I error of 0.05, we accepted that only a large treatment effect of sacubitril/valsartan in comparison to standard therapy would have been significantly detectable in terms of change in peak VO_2_ from baseline, in patients affected by HFrEF.

To be included in the study, participants had to meet the following criteria: (a) have a left ventricular ejection fraction (LVEF) < 40%; (b) have a stationary New York Heart Association (NYHA) functional class of at least II; (c) capability to carry out a valid CPET, and d) have undergone prior treatment with an ACE inhibitor or AR blocker.

Exclusion criteria for the patients that met the inclusion criteria were: (1) hospitalization 90 days prior to evaluation due to HF, (2) myocardial revascularization 180 days prior to the evaluation, (3) concomitant initiation during study follow-up or in the previous 6 months of cardiac resynchronization therapy (CRT) and/or percutaneous mitral valve treatment, (d) had congenital heart disease, (e) failure to execute CPET, and (f) severe renal/hepatic impairment or medical history of tumors.

All patients provided informed consent, and the research ethics committee approved the protocol in accordance with the principles of the Declaration of Helsinki and national regulations.

At each visit, baseline, and follow-up (median time: 16 months; IQ range: 11.5–22), we collected demographic information, medical history, vital signs, 12-lead electrocardiogram, cardiopulmonary exercise testing, standard laboratory data, pharmacological treatments information, and echocardiographic parameters. Doses of Sac/Val were prescribed and titrated to the maximally tolerated doses, according to established recommendations [14]. The primary end-point consisted of the changes from baseline in peak VO_2_ (adjusted to body weight). The secondary end-point included changes in ventilatory efficiency during exercise (VE/VCO_2_ slope), in percent predicted VO_2_, and in main echocardiographic (ejection fraction, left ventricle and left atrium volumes, E/e’ ratio, tricuspid annular plane systolic excursion, systolic pulmonary artery pressure) and hemodynamic (systolic blood pressure) parameters.

### 2.2. Cardiopulmonary Exercise Testing

All CPETs were conducted on a cycle ergometer, with the pedaling rate set to 60 rpm. A ramp exercise protocol was systematically followed, with the workload starting at 10 watts for a warm-up period of 2 min and increasing by 6 watts every 60 s thereafter. The patients were encouraged to exercise to the point of feeling unable to continue due to dyspnea or fatigue. The peak VO_2_ was calculated by determining the highest 30 s average within the final minute of the exercise, as recommended by Mezzani et al. [15]. To assess the ventilatory efficiency, we calculated the relationship between minute ventilation and carbon dioxide production (VE/VCO_2_ slope) over the entire exercise duration, as previously recommended [16].

The percentage of predicted VO_2_ represented the achieved peak VO_2_ adjusted for age, weight, and height and expressed as a percentage. We used the equations by Wasserman and Hansen to measure the percentage of predicted VO_2_ [17].

### 2.3. Echocardiographic Measurements

To perform echocardiographic examinations, a 3.5 MHz monoplane ultrasound probe of Vivid E-9 (GE-Vingmed Ultrasound, Horten, Norway) was used, following international guidelines [18]. To avoid bias, two expert operators blinded to clinical data assessed all the parameters offline. LVEF was calculated by the Simpson biplane method according to the following formula: LVEF = [left ventricular end-diastolic volume (LVEDV)-LV end-systolic volume (LVESV)]/LVEDV × 100 as the mean of two measures in four and two apical chambers. A biplane method was used for left atrial volume (LAV) assessment, as well. For the evaluation of early-diastolic filling (E), in the apical long-axis view, the pulsed-wave Doppler sample volume was placed at the extremeness of the tenting area of the mitral valve. In the apical 4-chamber view using Tissue Doppler Imaging (TDI), mean e’ was assessed in the basal inferoseptal and lateral LV region. Consequently, the ratio of mitral E peak velocity and averaged e’ velocity (E/e’) was calculated. Furthermore, by sampling the systolic trans-tricuspid pressure gradient, calculated by the modified Bernoulli equation, the tricuspid regurgitant jet velocity was determined. Subsequently, the systolic pulmonary artery pressure (sPAP) was calculated from the sum of the tricuspid regurgitant jet velocity with the estimated right atrial pressure, according to inferior vena cava dimension and inspiratory collapsibility. Finally, in the apical four-chamber view, by aligning the M-mode linear cursor to the lateral tricuspid annulus and evaluating the tricuspid annular plane systolic excursion (TAPSE), the right ventricular function was determined.

### 2.4. Statistical Analysis

Continuous variables were reported as mean ± standard deviation or median and interquartile (IQ) range if the data did not follow a normal distribution. Numbers and percentages were used for categorical variables. To check for normal distribution, the Shapiro–Wilk test was used. Baseline characteristics were analyzed using either the Student *t*-test or the Mann–Whitney test for parametric or nonparametric variables, respectively. To assess categorical variables, the chi-square and Fisher’s exact tests were used. For data analysis, a two-way repeated measures ANOVA was employed, with time (baseline, follow-up) and treatment (Sac/Val vs. standard optimal therapy) as factors. In the case of nonparametric variables, a Friedman test was applied. Bonferroni post hoc correction was applied when a significant time × treatment interaction was found. For all tests, a *p*-value <0.05 was considered statistically significant. Statistical analysis was performed using SPSS software version 23.0 (SPSS Inc., Chicago, Illinois) and R version 4.0.5 (R Foundation for Statistical Computing, Vienna, Austria) [19]. Sample size analysis was performed considering the main statistical study analysis, ANOVA with repeated measures and between factors difference, with the G-power 3.1 software.

## 3. Results

At baseline, demographic and clinical data, according to the main treatment (Sac/Val vs. control group), are described in Table 1, while echocardiographic and cardiopulmonary test data are depicted in Table 2. We did not observe significant differences between the two study groups at baseline.

**Figure 1:**
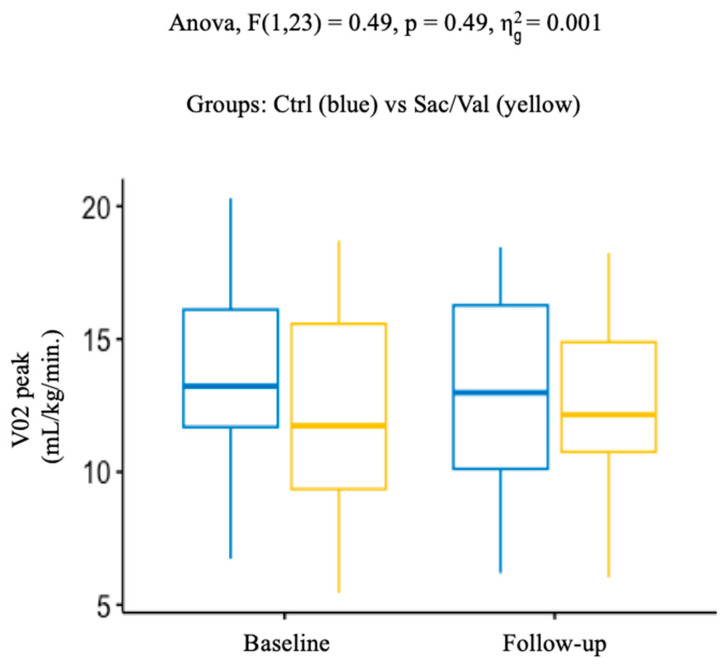
Changes of peak VO_2_, from baseline to follow-up, in the control group (blue) and the Sac/Val group (yellow) (*p* 0.49). VO_2_: oxygen consumption.

**Figure 2:**
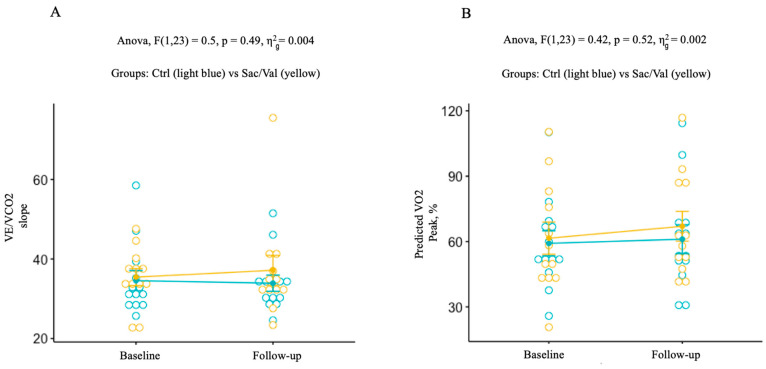
**Figure 2**. (**A**) Changes of VE/VCO_2_ slope, from baseline to follow-up, in the control group (blue) and in the Sac/Val group (yellow) (*p* 0.49). (**B**) Changes of predicted peak VO_2_ from baseline to follow-up in the control group (blue) and in the Sac/Val group (yellow) (*p* 0.53). VE/VCO_2_: minute ventilation to carbon dioxide production; VO_2_: oxygen consumption.

Similarly, we did not observe any significant differences during the follow-up, in terms of main CPET parameters, between the Sac/Val and the control group (Table 3, and Figure 1 and Figure 2A,B). Primary outcome analysis revealed, indeed, no differences in mean values of peak VO_2_ corrected for body weight between baseline and follow-up: Sac/Val 12.2 ± 4.6 and 12.7 ± 3.3 vs. 13.1 ± 4.2 and 13.0 ± 4.2 mL/kg/min, in the control group; *p* = 0.49. During the follow-up, no significant treatment differences were observed for changes in VE/VCO_2_: Sac/Val 35.4 ± 7.4 and 37.2 ± 13.1 vs. 34.6 ± 9.1 and 34.0 ± 7.3 in the control group; *p* = 0.49, as well as for predicted peak VO_2_: Sac/Val 61.5 ± 25.7 and 67.0 ± 23.7 vs. 59.2 ± 20.7 and 61.1 ± 23.9 %; *p* = 0.53. It is noted that, despite the lack of statistical significance in the other secondary outcomes analyzed, we observed a trend of improvement in main echocardiographic parameters in the Sac/Val group (Table 3). Moreover, the Sac/Val group showed a trend towards lower systolic blood pressure values during the follow-up (Table 3).

During the follow-up, none of the patients in the Sac/Val group interrupted the treatment; however, only three patients were able to tolerate the maximum dose. No deaths or rehospitalizations were recorded during the study.

## 4. Discussion

In our study, we found no significant differences between the Sac/Val and control groups in various measures of CPET. Although we did observe from the baseline a small improvement in peak VO_2_ in the Sac/Val group, it was lower than the clinically meaningful change of 1.5 mL/min/kg [20,21]. According to the literature, as shown by the HF-ACTION trial, even modest increases as low as 6% in peak VO_2_ can be associated with better clinical outcomes in HF patients (NYHA class II–IV) over a 3-month period [22]. In the Sac/Val group, we observed only a 4% increase between baseline and follow-up; however, the longer follow-up period considered in our analysis, along with the small sample size, could represent valuable reasons for such a small variation.

Currently, the CPET is considered the ‘gold standard’ for assessing functional capacity in HFrEF patients [23,24,25]. Few studies have investigated the effects of Sac/Val on improving the exercise capacity of patients affected by HFrEF, with discordant results [4,5,6,26,27,28]. Positive effects of Sac/Val on exercise capacity were often observed in retrospective, single-arm studies during short follow-up periods. Our study, with a longer follow-up period, confirms the lack of Sac/Val effects on exercise capacity, similar to the most recent trials [5,6,28]. Although Sac/Val is superior when compared with enalapril in reducing mortality and morbidity [1], it may have a limited impact on improving exercise capacity in patients with HFrEF. It should be noted that even other HF pharmacotherapies showed no benefit in improving exercise capacity [29]. As pointed out in the recent trial by Halle et al. [6], a possible explanation of our results is that the mean baseline values of VO_2_ peak in the Sac/Val and the control group were, respectively, 12.2 ± 4.6 and 13.1 ± 4.2 mL/kg/min, indicative therefore of a very limited exercise capacity, potentially refractory to further improvement. However, even with better baseline values of VO_2_ peak (19 mL/Kg/min), a recently published study showed similar negative results [5].

The positive trends showed in terms of main echocardiographic findings in the Sac/Val group during the follow-up means that our population, despite being limited in terms of sample size, is still representative of a real-world population of patients affected by HFrEF. The absence of significant results in both primary and secondary end-points, despite a longer follow-up, may also be related to the study design, where the timings of data entry and follow-up visits are different for each patient. Moreover, a dose effect cannot be ruled out as a possible further cause of unsuccessful improvement in CPET parameters (only three patients in our study achieved the maximum Sac/Val dose).

## 5. Limitations

There are several limitations to our study that should be acknowledged, which may restrict the generalizability of our results. First, we evaluated a small sample size in a retrospective, single-center study. In addition, the effects of Sac/Val on surrogate markers of efficacy (such as N-terminal pro-B-type natriuretic peptide levels) and functional capacity (NYHA class) were not evaluated [30]. At baseline, 74% of our patients were in NYHA class II. It is possible that an analysis performed in more severe HF patients (Class III) may bring different results. A larger sample size is required to increase the robustness of the results. However, it is challenging to identify a control group since the PARADIGM-HF established the long-term superiority of Sac/Val over enalapril in reducing the risk of cardiovascular death or HF hospitalization and all-cause death. Therefore, despite several limitations, our study provides a real-world population, including a control group and an extended follow-up period, thus supporting our findings.

## 6. Conclusions

After a median follow-up period of 16 months, there was no significant benefit of Sac/Val on peak VO_2_ and other measures of CPET compared with standard optimal therapy in patients with HFrEF.

## Figures and Tables

**Table 1:** Baseline characteristics of study population according to treatment.

Variables	Sacubitril/Valsartan (n = 12)	Control Group (n = 13)	*p* Value
Demographic and clinical data			
Age (years)	66.1 ± 7.9	60.8 ± 7.4	0.1
Female Gender N (%)	2 (16.7)	2 (15.4)	0.9
BMI (Kg/m^2^)	28.4 ± 4.3	27.4 ± 3.0	0.5
SBP (mmHg)	110 (110–130)	115 (110–125)	0.8
HR (beats/min)	70 (60–74)	60.5 (57.2–68.5)	0.2
NYHA Class			
2	8 (66.7)	9 (69.2)	0.6
3	4 (33.3)	3 (23.1)	
4	0	1 (7.7)	
Laboratory data			
Haemoglobin (g/dL)	14.7 (11.8–15.5)	14.3 (10.6–14.8)	0.3
Fasting glucose (mg/dL)	110 (92–121)	103 (93.5–111.5)	1
Creatinine (mg/dL)	1 (0.9–1.19)	1.06 (0.94–1.21)	0.7
Potassium (mEq/L)	4.36 ± 0.8	4.4 ± 0.74	0.8
eGFR (mL/min)	70.1 ± 20.6	72.5 ± 32.0	0.8
LDL (mg/dL)	86.4 ± 32.0	87.5 ± 33.4	0.9
Tryglicerides (mg/dL)	156.5 ± 74.3	104.7 ± 36.7	0.1
Comorbidities			
Coronary artery disease N (%)	8 (66.7)	5 (38.5)	0.2
Primitive dilated cardiomyopathy N (%)	3 (25)	6 (46.2)	0.4
Hypertension N (%)	10 (83.3)	9 (69.2)	0.6
Dyslipidemia N (%)	12 (100)	11 (84.6)	0.5
Atrial fibrillation N (%)	5 (41.7)	2 (15.4)	0.2
Diabetes N (%)	5 (41.7)	2 (15.4)	0.2
Chronic Kidney Disease N (%)	6 (50)	8 (61.5)	0.7
Chronic obstructive pulmonary disease N (%)	0	5 (38.5)	**0.04**
Thyroid disorders N (%)	4 (33.3)	2 (15.4)	0.4
Previous implantation of ICD/CRT N (%)	11 (91.7)	10 (76.9)	0.6
Medications N (%)			
β-blockers (bisoprolol)	12 (100)	13 (100)	-
Furosemide	11 (91.7)	12 (92.3)	0.95
Mineralcorticoid receptor antagonist (MRA)	11 (91.7)	12 (92.3)	0.95
Medications dose (mg)			
Bisoprolol	2.5 (2.5–3.75)	2.5 (1.25–4.37)	0.79
Furosemide	50 (25–68.7)	50 (25–75)	0.84
MRA	37.5 (25–50)	25 (25–50)	0.63

BMI: body mass index; SBP: systolic blood pressure; HR: heart rate; NYHA: New York Heart Association; eGFR: estimated glomerular filtration rate; LDL: low-density lipoproteins; ICD: implantable cardioverter defibrillator; CRT: cardiac resynchronization therapy; MRA: mineralocorticoid receptor antagonist. We have reported in bold the statistically significant *p*-value.

**Table 2:** Baseline echocardiographic and cardiopulmonary exercise test data, according to treatment.

Variables	Sacubitril/Valsartan (n = 12)	Control Group (n = 13)	*p* Value
Echocardiographic parameters			
LVEF (%)	30.7 ± 4.0	32.3 ± 5.1	0.4
LVEDVi (mL/m^2^)	116.0 ± 36.9	129.6 ± 32.4	0.4
LVESVi (mL/m^2^)	80.6 ± 27.9	86.0 ± 33.7	0.7
TAPSE (mm)	17.5 (16.2–21.2)	18 (16–21.5)	0.8
sPAP (mmHg)	32.1 ± 8.7	39.2 ± 9.6	0.1
LAVi (mL/m^2^)	44.2 ± 15.4	46.8 ± 15.6	0.7
E/e’ ratio	16.0 ± 7.5	15.2 ± 9.5	0.9
Cardiopulmonary exercise test data			
PeakVO_2_ (mL/kg/min)	12.2 ± 4.6	13.1 ± 4.2	0.6
VE/VCO_2_ slope	35.4 ± 7.4	34.6 ± 9.1	0.8
Predicted peak VO_2_, %	61.5 ± 25.7	59.2 ± 20.7	0.8

LVEF: left ventricular ejection fraction; LVEDVi: left ventricular end-diastolic volume index; LVESVi: left ventricular end-systolic volume index; E: early-wave transmitral diastolic velocity; e’: early-diastolic velocity at tissue Doppler imaging; TAPSE: tricuspid annular plane systolic excursion; sPAP: pulmonary artery systolic pressure; LAVi: left atrial volume index; VO_2_: oxygen consumption; VE/VCO_2_: minute ventilation to carbon dioxide production.

**Table 3:** Primary and secondary outcomes.

Variables	Baseline	Follow-Up	*p* Value
PRIMARY OUTCOME			
PeakVO_2_ (mL/kg/min)			
Sac/Val	12.2 ± 4.6	12.7 ± 3.3	0.49
Control group	13.1 ± 4.2	13.0 ± 4.2	
SECONDARY OUTCOMES			
VE/VCO2 slope			
Sac/Val	35.4 ± 7.4	37.2 ± 13.1	0.49
Control group	34.6 ± 9.1	34.0 ± 7.3	
Predicted VO_2_ peak (%)			
Sac/Val	61.5 ± 25.7	67.0 ± 23.7	0.53
Control group	59.2 ± 20.7	61.1 ± 23.9	
LVEF (%)			
Sac/Val	30.7 ± 4.0	36.1 ± 4.8	0.06
Control group	32.3 ± 5.1	33.1 ± 7.8	
LVEDVi (mL/m^2^)			
Sac/Val	116.0 ± 36.9	80.3 ± 19.1	0.09
Control group	129.6 ± 32.4	125.0 ± 37.2	
LVESVi (mL/m^2^)			
Sac/Val	80.6 ± 27.9	51.5 ± 15.8	0.08
Control group	86.0 ± 33.7	89.2 ± 31.0	
E/e’ ratio			
Sac/Val	16.0 ± 7.5	13.0 ± 10.0	0.07
Control group	15.2 ± 9.5	13.9 ± 10.0	
LAVi (ml/m^2^)			
Sac/Val	44.2 ± 15.4	42.8 ± 5.3	0.7
Control group	46.8 ± 15.6	43.8 ± 14.7	
TAPSE (mm)			
Sac/Val	17.5 (16.2–21.2)	20 (19–22)	0.4
Control group	18 (16.0–21.5)	20.5 (17.8–22.2)	
sPAP (mmHg)			
Sac/Val	32.1 ± 8.7	33.9 ± 10.4	0.17
Control group	39.2 ± 9.6	36.7 ± 12.3	
SBP (mmHg)			
Sac/Val	110 (110–130)	100 (925–110)	0.07
Control group	115 (110–125)	110 (100–125)	

Sac/Val: sacubitril/valsartan; VO_2_: oxygen consumption; VE/VCO_2_: minute ventilation to carbon dioxide production; LVEF: left ventricular ejection fraction; LVEDVi: left ventricular end-diastolic volume index; LVESVi: left ventricular end-systolic volume index; E: early-wave transmitral diastolic velocity; e’: early-diastolic velocity at tissue Doppler imaging; LAVi: left atrial volume index; TAPSE: tricuspid annular plane systolic excursion; sPAP: pulmonary artery systolic pressure; SBP: systolic blood pressure.

## Data Availability

Data available on request.
